# A Simple Method to Detect Recovery of Glomerular Filtration Rate following Acute Kidney Injury

**DOI:** 10.1155/2014/542069

**Published:** 2014-05-27

**Authors:** John W. Pickering, John Mellas

**Affiliations:** ^1^Department of Medicine, University of Otago Christchurch, P.O. Box 4345, Christchurch 8140, New Zealand; ^2^St. Mary's Health Center, Department of Internal Medicine, 6420 Clayton Road, St. Louis, MO 63117, USA

## Abstract

In acute kidney injury (AKI), elevated plasma creatinine is diagnostic of an earlier loss of glomerular filtration rate (GFR) but not of the concomitant GFR. Only subsequent creatinine changes will inform if GFR had already recovered or not. We hypothesized that the creatinine excretion rate to production rate ratio would provide this information. A retrospective analysis of 482 critically ill patients from two intensive care units (ICU) is shown. Plasma creatinine was measured on ICU entry and 12 hours later. Four-hour creatinine excretion rates (*E*) were measured on entry. Creatinine production rates were estimated (*eG*). The ability of the ratio *E*/*eG* to predict a decrease in plasma creatinine concentration, identify recovered AKI (≥0.3 mg/dL decrease), and predict AKI (≥0.3 mg/dL increase) was assessed by the area under the receiver operator characteristic curves (AUC). There was a linear relationship between reduced creatinine concentration and *E*/*eG* (*r* ^2^ = 0.15; *P* < 0.0001). *E*/*eG* predicted a decrease in creatinine (AUC 0.70 (0.65 to 0.74)), identified recovered AKI (0.75 (0.67 to 0.84)), and predicted AKI (0.80 (0.73 to 0.86)). A ratio of the rates of creatinine excretion to estimated production much less than 1 indicated a concomitant GFR below baseline, whereas a ratio much more than 1 indicated a recovering or recovered GFR.

## 1. Introduction

An apparent increase in serum creatinine is often the first indication of acute kidney injury (AKI) facing a clinician. Because the response of creatinine to changes in glomerular filtration rate (GFR) is delayed [1], a clinician cannot be certain if, at the time of the serum creatinine measurement, GFR has recovered or is still depressed. In the absence of real-time GFR measurements, subsequent serum creatinine measurements are needed to identify what had happened to GFR at previous time points. This temporal delay in ascertaining kidney function data means treatment for AKI may be applied where unnecessary or not applied when necessary, and drug dosing will be less accurate. In many cases in emergency departments or the intensive care unit, there is no available prehospitalization serum creatinine by which to estimate normal renal function. In such a situation, no estimate of a change in creatinine clearance can be made as has been suggested [2]. Furthermore, the duration of elevation of creatinine is associated with worse outcomes [3], but there is no current clinically available means to predict continued elevation. Over the past decade, considerable effort has gone into finding early markers of AKI with the emphasis being on structural injury biomarkers. In heterogeneous populations in the intensive care, in particular, these investigations have yet to identify good predictive biomarkers and suffer from having to compare apples (structural biomarkers) with oranges (creatinine as a surrogate of functional changes). There is, though, still progress to be made by considering creatinine kinetics. We consider here a novel methodology for predicting the direction of creatinine changes.

Under steady state conditions, the creatinine production rate equals the creatinine excretion rate. If production exceeds excretion, then most likely filtration is impaired compared with what is normal for the patient. If excretion exceeds production, then most likely an excess of creatinine is now being excreted and GFR is greater than the nadir GFR that resulted in the excess creatinine. We hypothesized that the ratio of a measured creatinine excretion rate to estimated creatinine production rate would provide meaningful clinical data on GFR changes. We tested this by comparing the ratio to subsequent changes in serum creatinine.

## 2. Materials and Methods

### 2.1. Creatinine Kinetics

Under steady state conditions, the rate of production of creatinine (*G*) equals the rate of excretion (*E*); therefore, the serum creatinine concentration (*C*) is constant. Excretion may be measured as the product of the urinary creatinine concentration (*U*) multiplied by the urinary flow rate (*V*).

The mass of creatinine in the body is equal to the plasma concentration multiplied by the volume of distribution (*D*) assuming that creatinine is evenly distributed throughout the volume of distribution. Because creatinine is water soluble, the volume of distribution is equal to the total body water. If the volume of distribution does not change or changes negligibly with time, then under nonsteady state conditions the rate of change of serum creatinine is

(1) $$\begin{array}{c} \frac{dC}{dt}=\frac{\left(G-UV\right)}{D}. \end{array}$$

**Figure 1:**
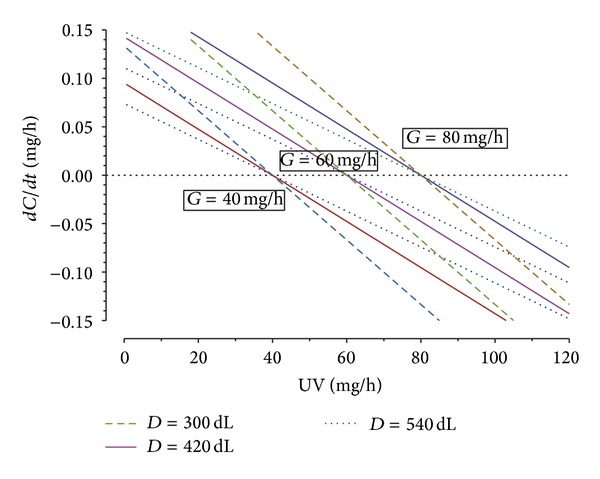
Rate of change of serum creatinine (*C*) as a function of excretion (UV) for different creatinine production rates (*G*) and volumes of distribution (*D*); see (1). *dC*/*dt* below zero indicates falling serum creatinine and above zero indicates increasing serum creatinine.

Equation (1) is plotted in Figure 1 for various values of *G* and *D*. *D* of 420 dL and *G* of 60 mg/h are used to represent a typical 70 kg male. Conceptually, if production exceeds excretion, creatinine will be expected to continue to rise, whereas if it is less than excretion it will fall. The ratio of excretion to production, *E*/*G*, therefore, determines the change in serum creatinine.

### 2.2. Clinical Evaluation

Patient data was from the EARLYARF study of 528 critically ill patients [4, 5]. All patients older than 15 years of age were eligible for inclusion. Principal exclusion criteria were not expected to survive 72 hours, not expected to remain in the ICU 24 hours, on renal replacement therapy or had already experienced a threefold rise in plasma creatinine from a known baseline, or already had a urine output <0.3 mL/kg/h for 6 or more hours. The EARLYARF study included a double-blind randomized control trial (RCT) of high-dose erythropoietin versus placebo to ameliorate or prevent AKI and an observational study of multiple biomarkers of AKI including short duration creatinine clearance. The study was the first of its kind to use a urinary biomarker of structural damage to the kidney to triage to an intervention and to use the relative average creatinine as an outcome. The RCT was negative [4] and subsequent biomarker analysis has included patients from both the intervention and nonintervention branches [5]. Urine and plasma creatinine samples were collected on entry to an intensive care unit (ICU), at 12 h and 24 h after entry and daily for up to 7 days. A four-hour urine volume was measured commencing at the time of sampling for the calculation of creatinine clearance [2]. Baseline creatinine was patient's preadmission normal creatinine value determined by review of clinical notes using the hierarchical approach described in [4]. 50% of the patients had pre-ICU creatinine enabling determination of a normal baseline; of the other 50%, the baseline was determined retrospectively from the last ICU value (56%), from minimum followup in 365 days (7%), or on admission creatinine (37%).

The formula of Bjornsson was used to estimate the creatinine production (*eG*) for each patient [6]:

(2) $$\begin{array}{c} \text{Males:}\;eG=\frac{\left(27-0.173\times \text{age}\right)\times \text{weight}}{24}\;\left(\text{mg}/\text{h}\right),\\ \text{Females:}\;eG=\frac{\left(25-0.175\times \text{age}\right)\times \text{weight}}{24}\;\left(\text{mg}/\text{h}\right), \end{array}$$

where age is in years and weight is in kilograms. A sensitivity analysis was conducted using alternate formulas to estimate creatinine production, namely, the 1976 formula of Cockcroft and Gault [7] and the 2011 nonphosphorous formula of Ix and colleagues [8].

The creatinine excretion rate on entry to the ICU (*E*) equalled the urine creatinine concentration on entry multiplied by the average urine flow rate over the first 4 hours. We plotted the ratio of *E*/*eG* against the measured change in plasma creatinine measured over the first 12 hours. We evaluated how well *E*/*eG* was correlated with the 12-hour change in creatinine and how well it predicted a decrease in creatinine using the area under the receiver operator characteristic curve (AUC). We also used the AUC to assess how well *E*/*eG* predicted an increase in plasma creatinine ≥0.3 mg/dL (AKI according to AKIN) or decrease in plasma creatinine ≥0.3 mg/dL (recovering AKI) within 12 hours. AUCs were compared using the approach of DeLong et al. [9].

We present 22 clinical examples of the use of *E*/*eG* by one author (JM) in an intensive care unit.

Matlab 2012b (MathWorks, Natick, MA, USA) and PRISM 6.0 (GraphPad, La Jolla, CA, USA) were used for statistical analyses.

## 3. Results

Four hundred and eighty-two patients had data enabling a calculation of changes in plasma creatinine over 12 hours and *E*/*eG* on entry to the ICU. Patient characteristics are given in Table 1.

**Figure 2:**
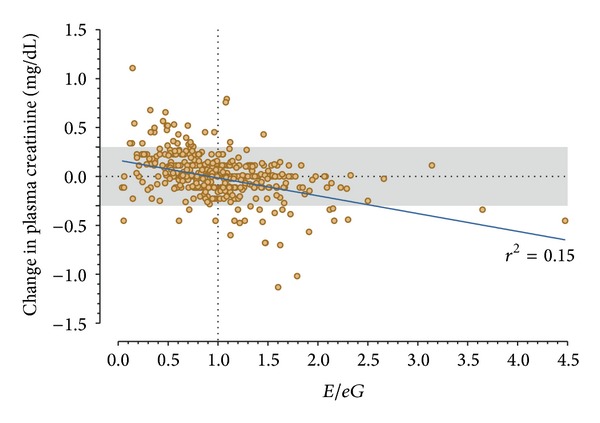
Change in plasma creatinine over 12 hours as a function of  *E*/*eG*. The solid line is the linear regression line. Patients above the shaded region had an increase of plasma creatinine ≥0.3 mg/dL (AKI). Patients below the shaded region had a decrease in plasma creatinine ≥0.3 mg/dL (recovering AKI).

**Figure 3:**
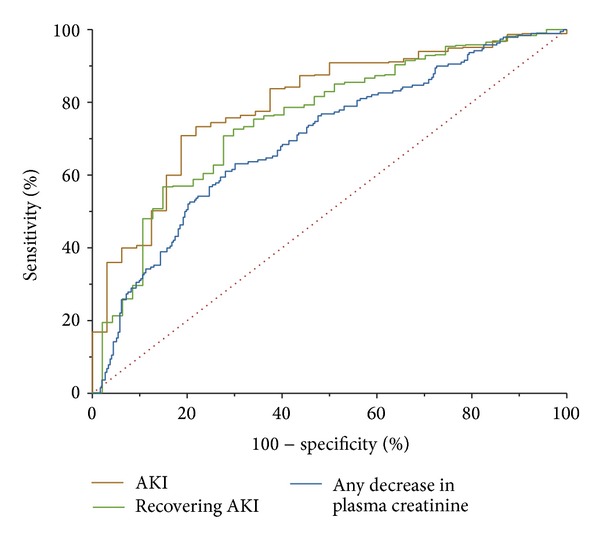
Receiver operator characteristic curves for the detection of (i) any decrease in plasma creatinine over 12 hours, (ii) recovery of AKI (≥0.3 mg/dL decrease), and (iii) AKI (≥0.3 mg/dL increase).

There was a linear relationship between change in creatinine and *E*/*eG* with a weak *r* ^2^ of 0.15 (*P* < 0.0001, Figure 2). Patients in the lower right quadrant of Figure 2 had experienced a fall in creatinine correctly predicted by an *E*/*eG* > 1, whereas patients in the upper left quadrant had experienced an increase in creatinine correctly predicted by an *E*/*eG* < 1. The AUC of *E*/*eG* to predict any decrease in plasma creatinine over 12 hours was 0.70 (95% confidence interval: 0.65 to 0.74). The AUC of *E*/*eG* to identify a recovered AKI (i.e., a decrease in plasma creatinine ≥0.3 mg/dL; *n* = 47) was 0.75 (0.67 to 0.84); see Table 2. The AUC of *E*/*eG* to predict AKI (i.e., an increase in plasma creatinine ≥0.3 mg/dL; *n* = 32) was 0.80 (0.73 to 0.86), Figure 3. The 90% sensitivity threshold of *E*/*eG* for AKI was 0.52; the 90% specificity was 1.08. The 90% sensitivity threshold of *E*/*eG* for recovered AKI was 1.55; the 90% specificity was 0.88.

### 3.1. Sensitivity Analysis for Creatinine Production Equation

The AUCs of *E*/*eG* to predict a decrease in plasma creatinine using Cockcroft and Gault and Ix formulas were not different from that using the Bjornsson formula; *P* = 0.18 and *P* = 0.92, respectively (Table 2).

### 3.2. Comparison to Other Metrics Associated with Kidney Function

Neither urine output in the first 4 hours nor the estimated production of creatinine predicted the decrease in plasma creatinine (Table 2). Creatinine clearance in the first four hours, plasma creatinine entry to the ICU, and the absolute and relative changes in plasma creatinine from baseline did predict the change but with AUCs much less than that of *E*/*eG* (Table 2). The AUC for the measured excretion of creatinine over four hours was 0.68 (0.63 to 0.73) and not statistically different from that of *E*/*eG* (*P* = 0.30).

### 3.3. Sensitivity Analysis: CKD Subgroup

Amongst 66 CKD patients, the AUC of *E*/*eG* to predict a decrease in plasma creatinine over 12 hours was 0.71 (0.58 to 0.83). The AUC of *E*/*eG* to predict a recovered AKI was 0.80 (0.59 to 1.0) and to predict AKI with increasing creatinine was 0.84 (0.71 to 0.97).

### 3.4. Clinical Examples

22 patients had measures of serum creatinine and 4- to 24-hour creatinine clearance at the time of nephrologist consultation (day 1) (Table 3). All 12 patients with an *E*/*eG* ratio of >1.55 recovered. The three patients with *E*/*eG* < 0.7 worsened, and two (both with *E*/*eG* = 0.2) required dialyses.

## 4. Discussion

The ratio of measured creatinine excretion to estimated creatinine production was associated with a subsequent change in plasma creatinine in a heterogeneous critically ill cohort and moderately predicted the direction of that change (AUC = 0.70). Patients already with recovered AKI could be identified (AUC = 0.75), and patients who would be diagnosed as AKI within 12 hours were diagnosed early (AUC = 0.80). An *E*/*eG* > 1.55 had 90% sensitivity for recovered AKI. The ratio performed equally well in a CKD only cohort. This method cannot distinguish between non-AKI patients and patients with AKI where the excretion and production are in a steady state some 24–72 hours after decrease of GFR. The AUCs for diagnosis of AKI are greater than that for urinary biomarkers of acute kidney injury in the same cohort, namely, *γ*-glutamyltranspeptidase (GGT), alkaline phosphatase (AP), neutrophil-gelatinase-associated lipocalin (NGAL), kidney injury molecule (KIM-1), and interleukin (IL-18) [5]. Even large well-defined cohorts in less heterogeneous populations, such as postcardiac surgery only, do not have greater AUCs [10]. Measuring excretion and calculating this ratio are also considerably cheaper than novel urinary biomarker tests and more readily available as these tests are not available in all jurisdictions. This suggests that, as a bedside tool, the ratio of *E*/*eG* may have value to the clinician to help determine whether or not GFR has been lost or is recovering. This is of particular assistance where there is no prior knowledge of a patient's normal (baseline) creatinine concentration which hinders a diagnosis of AKI on admission. The recognition that the duration of elevated creatinine, not merely its peak value, is associated with mortality [3, 11] means that *E*/*eG* may be used as an early identifier of risk.

Theoretically, an excretion to generate rate ratio of *E*/*G* < 1 is an evidence of a prior loss of GFR without subsequent recovery. An *E*/*G* = 1 suggests that a steady state has been reached. This may follow a loss of GFR or may indicate no change in GFR at all. An *E*/*G* > 1 suggests that excess creatinine is being excreted due to a recovery of GFR following an earlier loss. Because excretion rate and plasma creatinine are measured, a creatinine clearance can also be determined. This provides the additional information the clinician needs to determine the extent of the continued loss of GFR where *E*/*G* < 1 if a prehospitalization normal creatinine is available, thereby allowing an estimate of baseline creatinine clearance. Where *E*/*G*≅1, a low measured creatinine clearance may suggest that a new steady state has been reached without recovery of GFR and a normal creatinine clearance suggests no loss of GFR. A short duration urine output collection is necessary where kidney function may be varying. Longer durations will not capture this variation and merely result in an average GFR over the time period. Nevertheless, in the clinical examples presented durations of 4 to 24 hours proved useable. On the other hand, too short durations may result in inaccurate data. While we have shown 4 hours to be feasible [2], it may be that shorter durations are also feasible. A two-hour urine output collection in a cohort of 725 critically ill patients was seen to relate to mortality [12] and durations as short as 30 minutes have been used to measure inulin or creatinine clearance [13]. Serial measures of *E*/*eG* along with creatinine clearance will inform the clinician of the response to therapy not only whether GFR has improved or not but also whether it has returned to approximately normal levels or not.

Following diagnosis of AKI, subsequent increases in plasma or serum creatinine are often described as worsening of renal function or movement to more severe changes. Similarly, falls in creatinine are often thought of in terms of recovery of renal function. Because of the delay in plasma creatinine changes, the concepts of worsening or recovery based on creatinine are misnomers. The ratio of excretion to production on the other hand is concurrent with plasma creatinine and provides immediate information on whether renal function has recovered or not. This may be combined with a measured creatinine clearance or estimated GFR using a kinetic estimate of GFR such as that recently suggested by Chen [14].

Urine output or creatinine clearance or changes in plasma creatinine from baseline were not good predictors of subsequent changes in direction of plasma creatinine. The creatinine excretion rate alone performed almost as well as *E*/*eG* to predict a reduction in creatinine. From a pragmatic perspective, the excretion rate may be a useful biomarker of recovery. However, it does not have the theoretical basis to be the way *E*/*eG* does. The lack of improvement in the AUC for the ratio compared to the estimated excretion rate may be because of the limitations of creatinine generation estimating equations. Further investigations in other cohorts may reveal if these equations are the primary limiting factor of the method. Also, the current cohort is too small to investigate if excretion rate alone performs as well as the ratio across all body sizes and shapes where creatinine production is likely to be different. For these reasons, and also to investigate where creatinine generation may change [15]; there is a need for the development of new methods to determine creatinine production.

While creatinine on presentation had a reasonable AUC (0.83) for the prediction of a >0.3 mg/dL reduction in creatinine, it is not a stand-alone diagnostic tool for recovery because unlike *E*/*eG* creatinine does not indicate direction of change. Nevertheless, it may be that adding creatinine to the ratio could improve diagnostic performance. We assessed if this idea was feasible using the integrated discrimination improvement (IDI) and risk assessment plots based on logistic regression models of calculated risk of recovery from AKI [16–18]. The average calculated risk (IDI for the event) of recovery increased by 0.13 (0.06 to 0.20) with the addition of plasma creatinine to the ratio. This is a meaningful difference and further studies should look to incorporate both the ratio and creatinine itself into a clinical prediction model.

In practice, there are uncertainties involved in the method due to the use of estimating equations, similar to the uncertainties arising due to the use of equations to estimate GFR in CKD. In this case, we used the creatinine production rate estimating equations of Bjornsson which were based on steady state creatinine excretion from 1145 patients from multiple studies including that of Cockcroft and Gault [7]. Bjornsson's equations had *r* ^2^ of 0.966 for females and 0.919 for males. Neither the estimating equation of Cockcroft and Gault nor the recent equation of Ix and colleagues improved the predictive performance of *E*/*eG*. Figure 1 illustrates that uncertainties in estimates of the rate of production in creatinine are linearly related to uncertainties in the estimated rate of change of serum creatinine and independent of the measured excretion rate. Uncertainties in the estimate of volume of distribution result in larger uncertainties in rate of change of serum creatinine the further the patient is from steady state. The latter may be relevant if large fluid boluses have been administered [15, 19].

Our demonstration measured changes in plasma creatinine over 12 h and a rate of excretion averaged over 4 hours. It is possible in some patients that some variables changed more rapidly than others. Variations in plasma creatinine of more than 0.3 mg/dL over 48 h are considered indicative of AKI [20]. We considered a decrease in creatinine of 0.3 mg/dL to be indicative of recovered AKI and an increase of 0.3 mg/dL over 12 hours to be AKI. Therefore, this does not preclude some patients with changes in creatinine between −0.3 and 0.3 mg/dL from being AKI because they have already reached a new steady state or because the rate of change in creatinine is insufficient to achieve a diagnosis over 12 hours. *E*/*eG* performed moderately well at diagnosing AKI or recovered AKI within 12 hours (i.e., predicting creatinine increases of ≥0.3 mg/dL or decreases of ≥0.3 mg/dL). The additional clinical cases presented illustrate that the methodology may be used practically within the ICU to good effect even with longer creatinine clearances. Most often a nephrologist is called for a consultation when creatinine is already elevated suggestive of AKI, making the task at this stage to decide if the patient is likely to recover without the need for dialysis or not.

Creatinine is not always produced at a constant rate. Recently, it was shown that, following cardiac arrest, the rate of production probably drops dramatically before recovering [15]. In an animal model creatinine production has been seen to be reduced by sepsis [21] and muscle wastage will gradually decrease creatinine production during illness [22]. Furthermore, the method relies on an estimate of creatinine production rather than a measure. Unfortunately, there is not a reliable measure of creatinine production measures in the nonsteady state. Urine creatinine itself may be secreted as well as filtered and may vary with GFR [23]. As with creatinine clearance, the assumption is that this secretion makes a negligible contribution to urinary concentration. While we used the spot sample urine creatinine of the EARLYARF study in our demonstration, it is possible that improved accuracy would be to use the average of spot samples taken before and after the collection period or the urine creatinine concentration from the urine collected during this period.

The use of an estimate of creatinine production, the potential changes in creatinine production, the confounding of urinary creatinine and urine, and assay uncertainties mean that ratios of *E*/*eG* close to one are of limited value in identifying trends in GFR. As a rule of thumb, many nephrologists do not react to a change in plasma creatinine of less than 10% because of uncertainties in two creatinine measures. Given that *E*/*eG* relies on one measure of plasma creatinine, one of urinary creatinine, and a measure of urinary volume, we suggest, conservatively, that an *E*/*eG* between 0.7 and 1.3 be treated cautiously.

## 5. Conclusions

A ratio of the rate of creatinine excretion to estimated creatinine production much less than 1 indicates a concomitant GFR below normal (or baseline), whereas a ratio much more than 1 indicates a recovering or recovered GFR. Combined with a short duration creatinine clearance measured at the same time, this ratio is a useful adjunct to the clinician's diagnostic arsenal. In particular, when the ratio is high (>1.55), the excess excretion over production most likely indicates recovery from acute kidney injury, whereas when it is low (<0.55), the excess of production over excretion indicates acute kidney injury. In both instances, this diagnosis is possible several hours before a change in creatinine is observed and may be made in the absence of a known baseline creatinine.

## Figures and Tables

**Table 1:** Demographic profile and clinical outcome.

Variable	Cohort (*n* = 482)
Age (years)	59.7 ± 17.5
Sex (female)	189 (39.2)
Weight (kg)	79.3 ± 19.3
APACHE II score	18.1 ± 6.4
On entry plasma creatinine (mg/dL)	1.00 (0.79–1.36)
Chronic kidney disease	66 (13.7)
Cardiac arrest	63 (13.1)
Sepsis	96 (19.9)
Mechanical ventilation	433 (89.8)
Dialysis in the ICU	12 (2.5)
Hospital mortality	73 (15.1)
Mortality at 1 year	111 (23)
Length of ICU stay (hours)	78 (44–180)

Data expressed as mean ± SD, *n* (%), or median (lower quartile–upper quartile). APACHE II score: acute physiological and chronic health evaluation II score. ICU: intensive care unit.

**Table 2:** AUC comparisons for detection of any decrease in plasma creatinine.

Variable	Detection of any decrease in plasma creatinine			Detection of >0.3 mg/dL decrease in plasma creatinine		
	AUC (95% CI)	Optimal cutpoint	*P* compared with *E*/*eG* (Bjornsson [6])	AUC (95% CI)	Optimal cutpoint	*P* compared with *E*/*eG* (Bjornsson [6])
*E*/*eG* (Bjornsson [6])	0.70 (0.65 to 0.74)	0.87	na	0.75 (0.67 to 0.84)	1.12	na
*E*/*eG* (Cockcroft and Gault [7])	0.69 (0.65 to 0.74)	0.89	0.18	0.75 (0.67 to 0.84)	1.18	0.91
*E*/*eG* (Ix et al. [8])	0.70 (0.65 to 0.74)	0.83	0.92	0.75 (0.67 to 0.83)	1.01	0.63
Urine output in first 4 h (mL)	0.52 (0.47 to 0.57)	271	<0.0001	0.59 (0.50 to 0.68)	273	0.0002
Creatinine clearance (mL/h)	0.59 (0.54 to 0.64)	60	<0.0001	0.50 (0.41 to 0.59)	83	<0.0001
Plasma creatinine on entry (mg/dL)	0.59 (0.54 to 0.64)	1.01	0.0056	0.83 (0.75 to 0.90)	1.13	0.119
Absolute change in plasma creatinine from baseline (mg/dL)*	0.62 (0.57 to 0.67)	0.09	0.050	0.83 (0.76 to 0.91)	0.26	0.084
Relative change in plasma creatinine from baseline (%)*	0.62 (0.57 to 0.67)	11	0.044	0.81 (0.74 to 0.89)	37	0.196
*eG* (Bjornsson [6]) (mg/h)	0.52 (0.47 to 0.58)	53	<0.0001	0.57 (0.48 to 0.66)	49	0.004
*eG* (Cockcroft and Gault [7]) (mg/h)	0.53 (0.47 to 0.58)	44	<0.0001	0.57 (0.48 to 0.66)	47	0.003
*eG* (Ix et al. [8]) (mg/h)	0.51 (0.45 to 0.56)	50	<0.0001	0.57 (0.48 to 0.65)	54	0.003
*E*: creatinine excretion (mg/h)	0.68 (0.63 to 0.73)	39	0.30	0.75 (0.67 to 0.83)	56	0.89

*Changes in plasma creatinine are between the preadmission baseline determined from clinical notes and the first creatinine measurement in the ICU.

**Table 3:** Clinical cases within the ICU.

Urine collection duration (h)	*E*/*eG*	Prediction	Day 1 creatinine (mg/dL)	Day 2 creatinine (mg/dL)	Day 3 creatinine (mg/dL)	Outcome
24	2.5	Recovery	4.28	3.32	2.63	Recovery
24	2.4	Recovery	3.61	2.71	1.63	Recovery
8	1.9	Recovery	2.64	1.9		Recovery
24	1.5	Possible recovery	2.14	1.95		No change
16	2	Recovery	4.34	2.62	1.73	Recovery
24	4.8	Recovery	5.28	3.27	1.67	Recovery
16	1.8	Recovery	2.97	2.63	1.86	Recovery
24	1.5	Possible recovery	3.21	2.45	1.93	Recovery
24	1.4	Possible recovery	2.51	2.33		No change
8	2.9	Recovery	3.16	1.56		Recovery
16	3	Recovery	2.15	1.39	1.07	Recovery
24	1.3	Possible recovery	3.89	2.9		Recovery
8	3	Recovery	8.37	5.88		Recovery
24	1.3	Possible recovery	1.73	1.47	1.25	Recovery
4	3.7	Recovery	12.39	9.96	8.86	Recovery
24	1.9	Recovery	5.53	4	3.14	Recovery
8	0.66	Worse	1.62	1.99	2.17	Worse
24	0.2	Worse	2.49	3.36	3.69	Dialysis
24	0.7	Possible worsening	2	3.2	3.8	Worse
24	2	Recovery	2.25	2	1.79	Recovery
24	3.2	Recovery	15.13	9.14	5.29	Recovery
8	0.2	Worse	1.95	2.59	3.96	Dialysis
